# Supplementary material for: Transgenerational Perpetuation of CHS Gene Expression and DNA Methylation Status Induced by Short Oligodeoxynucleotides in Flax (Linum usitatissimum)
Source: Int J Mol Sci. 2019 Aug 16;20(16):3983. doi: 10.3390/ijms20163983 (PMC6719086; doi:10.3390/ijms20163983)
Supplement: Supplementary file 1 [file ijms-20-03983-s001.pdf]

1. Supplementary Materials

1.1. The stabilization of the epigenetic changes in the ODN6- treated flax after multiple passages in the in vitro conditions

Due to the low level of transfer to the next generation of epigenetic changes induced by ODN6, the stability of the obtained changes was monitored in the *in vitro* culture during passages.
In the *in vitro* cultured ODN6- treated flax the level of the CHS gene expression was assessed (Fig. S1A). Similarly to the primarily observed changes, after multiple passages in the ODN6 - treated flax the repression of the *CHS* was maintained (RQ = 0.7, in the comparison to the control set as 1).
The total methylation of the *in vitro* cultured treated with ODN6 flax was determined. Similarly to the observed originally changes, the total methylation of cytosines was elevated in the comparison to the control (Fig. S1B).

The expression of genes involved in the epigenetic modifications was investigated in the *in vitro* cultured ODN6 treated plants (Fig. S1C). The results have shown that the "epi-genes" are repressed after *in vitro* cultivation, as it was observed after 24h since the moment of incubation with oligonucleotides. Only the gene expression of *CMT3* was not repressed, at the control level (set as 1).

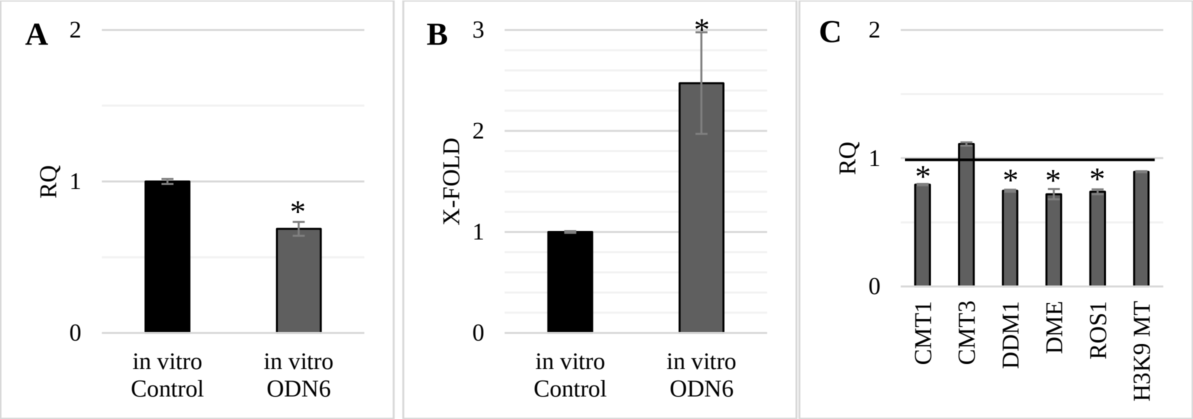

Figure S1. CHS gene expression (A), total genomic methylation (B) and expression of genes involved in epigenetic modifications (C) in the *in vitro* cultivated ODN6 treated plants. Plants after incubation with ODN were cultivated in the *in vitro* conditions and passaged every 1 to 2 moths into new medium during 3 years. (A) The expression of two CHS genes, *LuCHS6* and *LuCHS7* was determined by the Real-Time PCR reaction. The values are referred to the reference gene expression actin. The Relative Quantity (RQ) presents the transcript level in the comparison to the control (set as 1, black). (B) The determination of total 5-methylcytosine in the analyzed plants was performed using a technical kit dedicated for total methylation assay (detailed description in Material and Methods section). The values are referred as x-fold in the comparison to the control (set as 1, black). (C) The expression of genes involved in epigenetic modifications was determined by the Real-Time PCR reaction. The transcript level of genes encoding following enzymes: methylases (CMT1 – chromomethylase 1, CMT3 – chromomethylase 3), demethylases (DME – DEMETER, ROS1 – repressor of silencing 1) and enzymes involved in chromatin methylation (DDM1 – decrease in DNA methylation 1, H3K9 MT – histone H3K9 methyltransferase) was presented. The values are referred to the reference gene expression actin. The Relative Quantity (RQ) presents the transcript level in the comparison to the control (set as 1, black). All presented data constitute the mean value ± SD from at least three independent experiments. The significance of the differences between each mean and control was determined by Student's t-test. Asterisk indicates $p < 0,05$

1.2. *The transcript level of genes encoding other than CHS enzymes involved in the phenylpropanoid biosynthesis pathway*

In the Figure S2 genes coding enzymes involved in the synthesis of phenylpropanoid compounds (other than CHS): phenylalanine ammonia (PAL), cinnamate 4-hydroxylase (C4H), 4-hydroxycinnnamoyl ligase: CoA (4CL), p-hydroxycinnomoCoA transferase: shikimic/quinonic acid (HCT), β-cumarate-3-hydroxylase (C3H). The transcript level of these genes were determined for plants treated with: ODN1 (Fig. S2A), ODN1 met (Fig. S2B) and ODN1 pto (Fig. S2C), after 10 days from the time of treatment.

For plants incubated with ODN1, no significant changes in gene expression for *4CL*, *C4H* and *C3H* was observed, in comparison to control. However, lower transcript levels were presented by genes encoding PAL and HCT, for both genes RQ = 0.8 (Fig. S2A). Any significant differences in the expression of phenylpropanoid metabolism genes was noted in plants incubated with ODN1 met (Fig. S2B). However concerning ODN1 pto, plants showed a significant increase in expression of analyzed genes. The most significant overexpression of the tested genes was reported for *HCT* (2.3-fold increase), *PAL* (2-fold increase) and *C4H* (1.7-fold increase). Other genes encoding 4CL and C3H have shown a 1.5-fold increase of the transcript level in the comparison to the control (Fig. S2C).

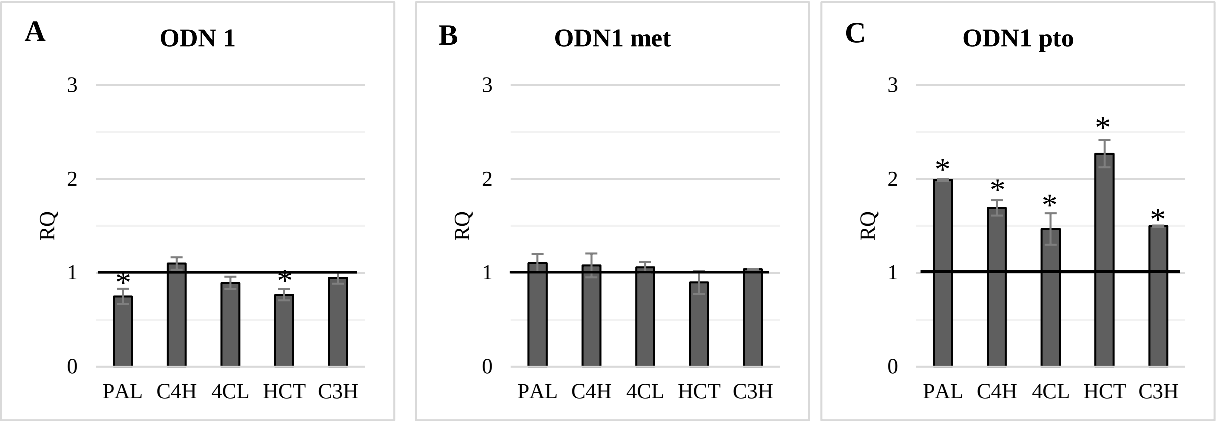

Figure S2. Transcript quantity derived from genes (other than CHS) involved in the synthesis phenylpropanoid compounds, determined in the plants treated with ODN1: unmodified (A), methylated (B) and tiophosphorylated (C). The expression level of genes encoding following enzymes was studied: PAL (phenylalanine ammonia-lyase), C4H (cinnamate-4-hydroxylase), 4CL (4-coumarate-CoA ligase), HCT (hydroxycinnamoyl transferase) and C3H (cinnamate-3-hydroxylase). The values are referred to the reference gene expression actin. The Relative Quantity (RQ) presents the transcript level in the comparison to the control (set as 1, black). All presented data constitute the mean value ± SD from at least three independent experiments. The significance of the differences between each mean and control was determined by Student's t-test. Asterisk indicates $p < 0,05$

Target gene or sequence		Primer forward	Primer reverse
**Reference gene**			
actin		5' CCGGTGTTATGGTTGGAAT 3'	5' TGTAGAAAGTGTGATGCCAAA 3'
**CHS gene expression**			
CHS (*LuCHS6* and *LuCHS7*)		5' CCCACGTAATATTCTGCACAAGTA 3'	5' GCGCCTCGATTGTTCTC 3'
LuCHS6		5' ACCATTGGGGGATAATATTGTTGG 3'	5' CTTCCCGGGCTCAAGCCTC 3'
LuCHS7		5' GCCATTGGGGGA TATTGTTGG 3'	5' CTTCCCGGGCTGGAGCCTT 3'
**-CCGG- motif (*LuCHS6*/*LuCHS7*)**			
coding	+552/+996	5' ACCAACTCACCATGCTCTTG 3'	5' GCGAAGTCGTCGTCACTAGG 3'
	+775/+1219	5' CGTAGTCTGGTGCCAGATCA 3'	5' CTCGAACAACGGCCTCTCT 3'
	+829/+1273	5' GGGGCCCAGACTATTGTACC 3'	5' CAATCACAATCGCCAACAAT 3'
**The expression of genes encoding enzymes involved in the epigenetic modifications**			
CMT1		5′ CAGATTTCGCTCCACAGTA 3′	5′ AGAAATGTCCCATTGCTCTAT 3′
CMT3		5′ AAAGGGTGCTAACTTCAGG 3′	5′ GACCAAATGGTTTAGACGATGT 3′
DME		5′ATGGCTACGGAGGCTACTTA 3′	5′ TGTTTCACCTGGTGTCCATA 3′
ROS1		5′ GCACTGAGAAGAAGTGCC 3′	5′ CTTAATGCGTGCTGCAAG 3′
DDM1		5′ GTGGTTAATATCTTTGTGGCAG 3′	5′ CATCCAATTTGCCAGAGTAG 3′
H3K9		5′ TGCCAAAGGTGTAAAGCTC 3′	5′ ATCCTTGCTTCGATTAGCC 3′

Table S1. Primers used in the Real-Time PCR reaction

ATGTCGACCATCACCGTTGATGAAGTACGCAAGGCTCAACGTGCACAAGGTCCCGCCACCGT
CCTTGCCATCGGAACCGCCACTCCTGCCAACTGCGTCGATCAAAGCACCTACCCTGATTACTA
CTTCCGTATTACCAACAGCGAGCACAAGACTGAGCTCAAGGAGAAGTTCAAGCGCATGTGTA
TGTTCATTCGTATTAATTGTTATTTCATTCTAGAAATTTTCATTTTTTGTTGCATTATTATTCATCT
ACGTTACGTATGTACAATCTGGAGATTGAAAAGCCCATGTTTGAAATTGTGTCCACAGCCCAG
ACCATCCTGCCCGATAGTGAAGGTGCTATCGACGGACACCTTCGGGAAGTGGGGTTGACTTTC
CACCTTCTGAAAGATGTCCCCGGGCTGATTTCGAAGAACATTGAGAAGAGCTTGGTGGAGGC
GTTTAAGCCGTTGGGGATATCGGACTGGAACTCGCTTTTCTGGATAGCTCATCCGGGTGGTCC
GGCGATTCTGGACCAAGTGGAGGCTAAGTTGAACCTCAAGGAGGAGAAACTGCGAGCCACG
AGGCAGGTTCTGGCTGATTATGGTAACATGTCGAGTGCTTGTGTGTTGTTCATATTGGATGAGA
TGAGGAAGAAATCTGTTGCGGATGGGTTGAACACTACTGGTGAAGGGCTTGATTGGGGGGTTC
TGTTTGGATTCGGGCCTGGACTCACTGTGGAGACTGTAGTTCTTCACAGTGTGGCTGTTTGA

Figure S3. Recognized gene fragment sequence of *LuCHS11 (*Acc. no AFSQ01012744) analyzed in this study.
